# Efficacy of Belimumab for active lupus nephritis in a young Hispanic woman intolerant to standard treatment: a case report

**DOI:** 10.1186/s12882-018-1066-3

**Published:** 2018-10-20

**Authors:** Francesco Fontana, Gaetano Alfano, Marco Leonelli, Caterina Cerami, Giulia Ligabue, Amelia Spinella, Giorgia Citriniti, Carlo Umberto Manzini, Clodoveo Ferri, Gianni Cappelli

**Affiliations:** 1 Surgical, Medical and Dental Department of Morphological Sciences, Section of Nephrology, University of Modena and Reggio Emilia, Modena, Italy; 2 Nephrology Dialysis and Transplant Unit, University Hospital of Modena, Modena, Italy; 3 Department of Medical and Surgical Sciences for Children and Adults, Section of Rheumatology, University of Modena and Reggio Emilia, Modena, Italy

**Keywords:** Lupus nephritis, Belimumab, Infectious complications, Case report

## Abstract

**Background:**

Lupus nephritis (LN) is a frequent severe complication of Systemic Lupus Erythematosus (SLE), especially in patients of non-Caucasian ethnicity. Induction treatment for LN consists in the combination of steroids plus a second agent (cyclophosphamide or mycophenolate mofetil) or, as a second-line, calcineurin inhibitors or Rituximab. Induction treatment for LN can be complicated by a series of side effects, the most severe being serious infections. Belimumab is a fully humanized monoclonal antibody that targets soluble B lymphocyte stimulator (BLyS), approved for treatment of serologically active SLE in addition to standard of care.

**Case presentation:**

A young Hispanic woman was diagnosed with SLE at the age of 15. After several immunosuppressive treatments for arthritic symptoms (high-dose steroids, mycophenolate mofetil, Rituximab, cyclophosphamide) leading to serious complications and scarce clinical improvement, she developed severe LN. Induction treatment with a combination of intravenous high-dose methylprednisolone and cyclophosphamide was started but, after few days, the patient developed cryptococcal meningitis. After institution of appropriate antifungal therapy, treatment with Tacrolimus was attempted but poorly tolerated by the patient and withdrawn. Eventually, Belimumab was initiated off-label as a last resource to treat LN. Belimumab was well tolerated by the patient and resulted in a rapid and marked improvement in clinical symptoms and reduction in proteinuria, serum complement levels and anti-dsDNA titer; of note, the patient developed no infectious complications.

**Conclusions:**

We report the case of a severe LN in a young Hispanic woman who did not respond to conventional and second-line induction therapies, due both to intolerance and to the development of serious infectious complications. Eventually, Belimumab was successfully added to steroids and was well tolerated by the patient, resulting in a marked improvement in clinical and biochemical parameters. We suggest that Belimumab should be considered as a potentially efficacious treatment in patients with LN who cannot tolerate conventional therapies.

## Background

Lupus nephritis (LN) develops in approximately one third of Systemic Lupus Erythematosus (SLE) patients, being most common severe lupus manifestation [1]. The frequency and severity of LN is increased in patients of non-Caucasian ethnicity [2]. Standard treatment of active proliferative LN involves an induction phase where the use of aggressive immunosuppression is advocated; regimens validated by large clinical trials comprise high dose steroids in combination with a second agent (cyclophosphamide or mycophenolate mofetil) [3]. Also, calcineurin inhibitors can be used as second-line treatment [4]. Although not validated by clinical trials, Rituximab can have a role in resistant cases [5]. Induction treatment for LN can be complicated by a series of side effects, the most severe being serious infections. After clinical improvement, induction treatment is usually followed by a maintenance phase, where usually low dose steroids plus an anti-proliferative drug (mycophenolate mofetil or azathioprine) are used [6]. The B lymphocyte stimulator (BLyS) is a survival factor that binds to specific receptors on B cells; BLyS inhibits apoptosis of B cells and promotes their proliferation and antibody production. Belimumab is a fully humanized monoclonal antibody that targets soluble BLyS and prevents BLyS from engaging its receptors on B cells. Belimumab is approved for treatment of serologically active SLE in addition to standard of care, and has been used in maintenance treatment after rescue induction treatment [7]; its use is not indicated in SLE complicated with LN, mainly because the trials which validated its use lacked inclusion of patients with renal involvement [8–10]. Recently, a systematic review of the literature merging data from large clinical trials and few case reports suggested that Belimumab can have a positive role in the treatment of LN [11].

## Case report

We present the case of a young Hispanic woman of Peruvian descent who was diagnosed with SLE in September 2015 at the age of 15 in her home country. She initially presented with arthritic symptoms and no cutaneous involvement; serum anti-nuclear antibodies (ANA) were positive at high titer, as were anti-double strand DNA antibodies (anti-dsDNA), and there was a marked reduction in serum complement fractions; a proteinuria of 0,6 g per day was documented, with normal serum creatinine. A kidney biopsy was not performed, and the patient was treated with 4 high dose steroid pulses plus one cyclophosphamide pulse and subsequent oral steroid tapering, oral hydroxychloroquine and angiotensin converting enzyme inhibitor. Of note, during hospitalization she developed pneumonia which required multiple unspecified antibiotic treatment. One month after, she was hospitalized at the Rheumatology clinic of our Hospital for revaluation; mycophenolate mofetil was introduced at the dose of 1 g per day, and steroid was maintained (at the dose of 1 mg/kg per day of prednisone). After 3 months she developed hypertransaminasemia, which prompted withdrawal of mycophenolate mofetil and hydroxychloroquine; other causes of hepatitis (namely viral and autoimmune) were excluded by means of specific serology; of note, low grade proteinuria persisted. In April 2016 a new course of immunosuppressive treatment with Rituximab at the dose of 375 mg/m^2^ was attempted; after the second weekly infusion the patient developed pneumonia requiring hospitalization; a CT scan of the chest showed tree-in-bud pattern in the inferior right lobe, and a bronchoalveolar lavage was performed, retrieving no isolates for bacterial (including mycobacteria), viral or fungal pathogens. The patient was treated with empiric antibiotic therapy and discharged after resolution of symptoms. She was again hospitalized in July 2016 for worsening of arthritic symptoms and nephrotic range proteinuria; central nervous system involvement was excluded by means of head MRI. SLE Activity Index (SLEDAI) was 38, a value consistent with severe flare of SLE. The patient was again treated with a single 300 mg cyclophosphamide intravenous pulse while maintained with oral prednisone 1 mg/kg per day; she immediately developed respiratory symptoms consistent with bronchial infection and received treatment with antibiotics and intravenous immunoglobulins with benefit; cyclophosphamide was then administered orally at 1 mg/kg per day. She was once more hospitalized 1 month after for nausea and headache; she had marked hypertransaminasemia, proteinuria and hematuria, with normal kidney function; cyclophosphamide was immediately withdrawn, and a liver biopsy showed evidence of iatrogenic damage; meanwhile, she also received 5 plasmapheresis treatments for the suspicion of autoimmune hepatitis. The patient constantly maintained ANA test positivity (measured by indirect immunofluorescence) and a very high level of anti-dsDNA (> 2000 U/ml, measured by fluorescent beads assay), and marked consumption of serum complement (C3 41 mg/dl [reference range 90–180], C4 8 mg/dl [reference range 10–40]).

**Fig. 1:**
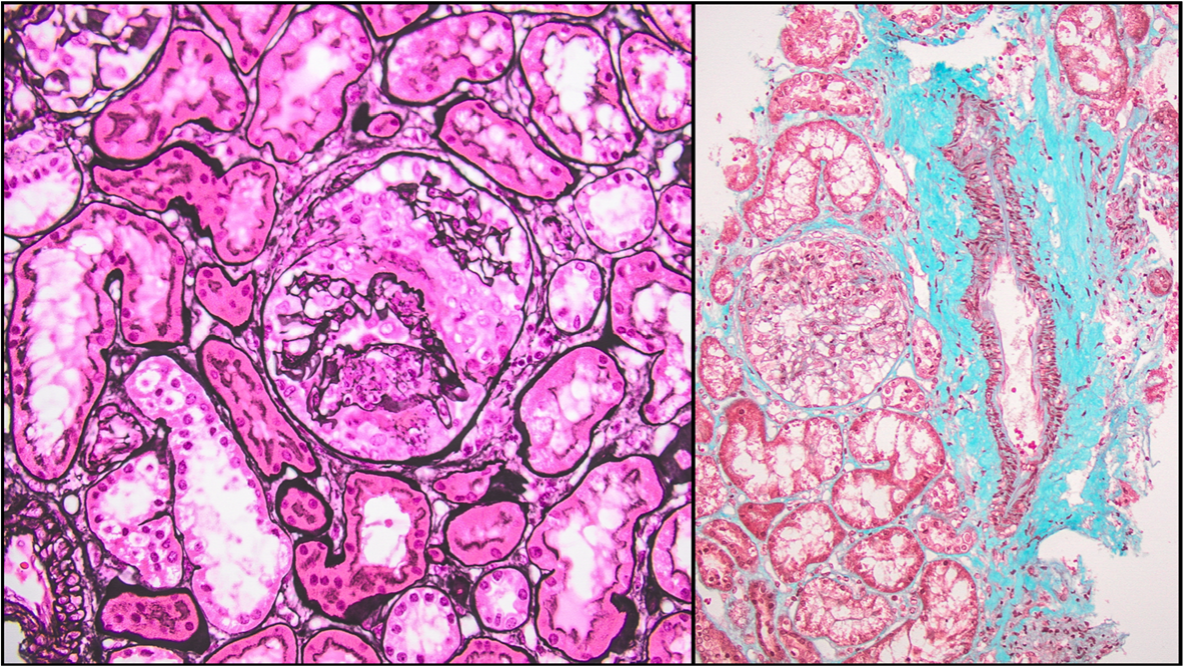
Kidney biopsy showing on light microscopy evidence of crescent formation in one glomerulus (left panel, PAS staining) and glomerular segmental endocapillary proliferation (right panel, Masson Trichrome staining)

**Fig. 2:**
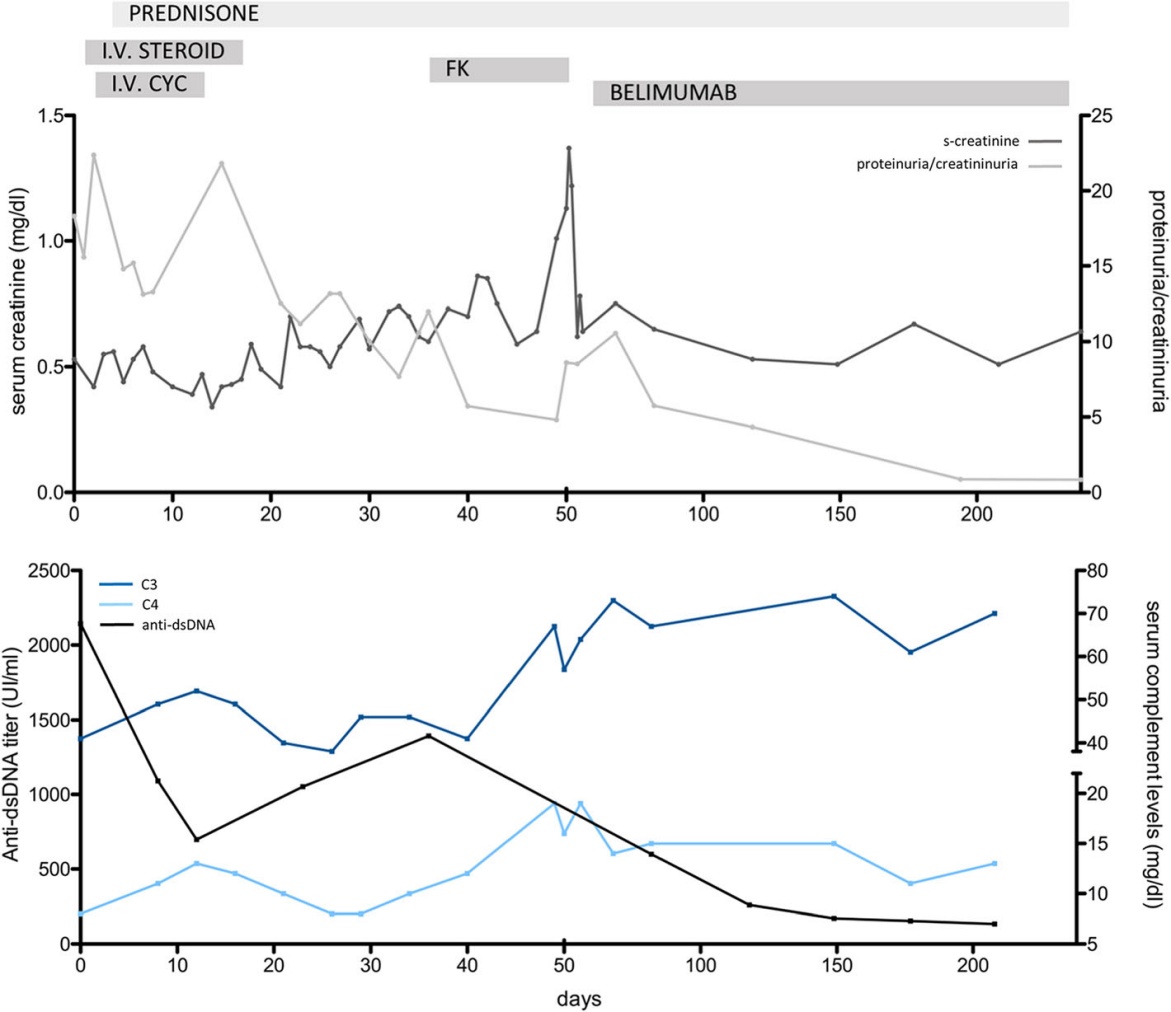
Trends of serum creatinine (top panel, dark-grey line), proteinuria/creatininuria (top panel, light-grey line), serum C3 (bottom panel, dark-blue line), serum C4 (bottom panel, light-blue line), anti-dsDNA titer (bottom panel, black line) from the date of kidney biopsy over time and through the different courses of Immunosuppressive therapies. I.v. steroid: three intravenous 500 mg methylprednisolone pulses; i.v. cyc: one intravenous 500 mg cyclophosphamide pulse; Fk: tacrolimus; prednisone: oral tapering prednisone

In September 2016 the patient was referred to the Nephrology unit for evaluation; physical examination was unremarkable except for facies lunaris and leg muscle hypotrophy related to long-time steroid use. A kidney biopsy was performed for the persistence of nephrotic-range proteinuria (proteinuria/creatininuria on a single void urine sample 18; serum albumin 3, 5 g/dl) associated with microhematuria (3+ on dipstick) and normal kidney function (serum creatinine 0, 5 mg/dl). Histological analysis revealed on light microscopy diffuse glomerular segmental necrosis with crescent formation (see Fig. 1), diffuse tubular vacuolization, diffuse interstitial edema and normal small-size arteries, and on immunofluorescence weak diffuse IgG, C3, kappa and lambda chain sub-endothelial deposits; the findings were consistent with diffuse segmental proliferative LN (class IV-S ISN-RPS classification [12]). Induction treatment was initiated with the administration of three pulses of intravenous steroid (Methylprednisolone 350 mg) and one pulse of intravenous cyclophosphamide (500 mg). A skin lesion in the tight was biopsied and cutaneous vasculitis consistent with SLE was diagnosed. Three days after discontinuation of intravenous immunosuppressive treatment, the patient developed high-grade fever with shivering and intense headache. A lumbar puncture was performed and liquor cultures resulted positive for Cryptococcus; treatment with liposomal amphotericin B and fluocytosine was initiated. After obtaining negative blood cultures initial treatment was subsequently switched to fluconazole. For persistence of nephrotic syndrome, after multidisciplinary discussion, treatment with a calcineurin inhibitor (tacrolimus) was attempted; unfortunately, administration of this drug resulted very poorly tolerated by the patient, who developed acute gastrointestinal symptoms with acute kidney injury stage 2 as per KDIGO guidelines [13], and treatment was discontinued. Given the very poor renal and overall prognosis for LN if left untreated, use of Belimumab as a last resource was deemed reasonable, although off-label. The patient was started on fortnightly Belimumab infusion at the dose of 10 mg/kg while maintained on prednisone at the dose of 12, 5 mg per day. After 1 month of treatment, Belimumab was switched to monthly administrations and steroid tapering was commenced. Belimumab was well tolerated by the patients and arthritic symptoms gradually improved; of note, the patients remained free from infections. Kidney function remained normal, and proteinuria and hematuria gradually decreased. Anti-dsDNA antibodies titer diminished rapidly and steadily; serum complement levels (which were already starting to decline before Belimumab initiation) normalized almost completely. Trends of serum creatinine, proteinuria/creatininuria, serum complement and anti-dsDNA are depicted in Fig. 2.

Presently, after 6 months of treatment with Belimumab, the patient is asymptomatic, with a serum creatinine of 0, 5 mg/dl, a proteinuria/creatininuria on single void urine sample of 0,87, 2+ hematuria on dipstick, C3 70 mg/dl and C4 13 mg/dl, anti-dsDNA 134 U/ml. Measured SLEDAI score is 10, consistent with mild SLE activity. Considered the fair improvement of the clinical picture, a slow steroid tapering is currently ongoing.

## Discussion

Treatment of active proliferative LN can be challenging, especially for patients who do not respond to first line treatments or those who develop severe infectious complications. In this subset of patients, substantial risk of progression to end stage renal disease must be weighted about the potentially fatal risk of infections. Before the introduction of immunosuppressive therapy, the prognosis of LN was extremely dismal [14]. Despite the great improvement in modern standard of care, the presence of LN is associated with an extremely high risk of end stage renal disease (ESRD) and still an increased risk of death; also, the presence of renal insufficiency is linked to lower health-related quality of life [2]. Premature atherosclerosis, possibly due to the chronic inflammatory state of SLE [15], is now recognized as one of the leading causes of morbidity and mortality in SLE [16]. Nevertheless, it should be kept in mind that a substantial contribution to mortality in LN patients is conferred by serious infections [16] (especially in under-developed countries) and active disease. Cryptococcal meningitis is the most commonly reported invasive fungal diseases in SLE [17, 18]; most commonly, invasive fungal infections present early in the disease course, are associated with high disease activity and high-dose steroid use, and bear a substantial risk for fatal outcome [17]. The diagnosis of invasive fungal infections may be complicated by the overlap of clinical characteristics with active SLE; in our case, the presence of meningeal symptoms was initially attributed to possible SLE involvement of the central nervous system; nevertheless, this last hypothesis was rapidly discarded an appropriate diagnostic procedure (lumbar puncture) was performed, demonstrating the presence of fungal meningitis. After appropriate antibiotic treatment was instituted, in accordance with patient’s will and after discussion with her mother we decided to insist on immunosuppressive treatment despite complications and poor tolerance aiming at minimizing the consistent risk of rapid progression to ESRD and of extra-renal manifestations of SLE. Two major randomized trials (BLISS-52 [8] and BLISS-76 [9]) validated the use of Belimumab in addition to standard of care for the treatment of active SLE; apart from demonstrating the clinical efficacy of Belimumab versus placebo, these studies showed that rates of serious adverse events, including infectious complications, did not differ substantially across groups. Moreover, post-hoc subgroup analyses of patients with LN in the two BLISS studies indicated greater renal disease improvements with Belimumab than with placebo [19]. Since data available at the moment suggested that infections do not seem to be a major concern in patients treated with Belimumab, and considering the available reports [20–22] of successful treatment of LN with Belimumab and the suggestions derived from the BLISS trials [11, 19], after retrieval of ethical committee approval we proceeded with this treatment in adjunction to steroids. Belimumab is so far well tolerated by our patients, with absence of notable side effects or adverse events. Belimumab resulted in a strong improvement in parameter related to disease activity and in a consistent reduction in proteinuria and microhematuria. Consistent with results of previously mentioned clinical trials [8, 9], Belimumab resulted in a fair reduction in SLEDAI score. We hope that in the near future ongoing clinical trials [23] can establish a clear role for Belimumab in the treatment of LN, and better identify those patients who may benefit the most from this treatment. Meanwhile, we suggest that Belimumab may be used off-label in that subset of patients with active LN who develop severe infectious complications after first and second-line conventional treatments for LN or who are intolerant to them.

## Conclusions

We report the case of a severe LN in a young Hispanic female, who was deemed untreatable with conventional and second-line induction therapies, due both to intolerance and to the development of serious infectious complications, among which cryptococcal meningitis. Eventually, Belimumab was successfully added to steroids and resulted in a marked improvement in clinical and biochemical parameters, with disappearance of arthritic symptoms, reduction in proteinuria and microhematuria, reduction of anti-dsDNA antibodies and normalization of serum complement at 6 months. We suggest that Belimumab should be considered as a potentially efficacious treatment in patients with LN who cannot tolerate conventional therapies.

## Abbreviations

ANA: Anti-nuclear antibodies
Anti-dsDNA: Anti-double strand DNA antibodies
ESRD: End Stage Renal Disease
LN: Lupus Nephritis
SLE: Systemic Lupus Erythematosus
SLEDAI: Systemic Lupus Erythematosus Activity Index
